# Development, comparison, and internal validation of prediction models to determine the visual prognosis of patients with open globe injuries using machine learning approaches

**DOI:** 10.1186/s12911-024-02520-4

**Published:** 2024-05-21

**Authors:** Mehrdad Motamed Shariati, Saeid Eslami, Nasser Shoeibi, Alireza Eslampoor, Mohammadreza Sedaghat, Hamid Gharaei, Siamak Zarei-Ghanavati, Akbar Derakhshan, Majid Abrishami, Mojtaba Abrishami, Seyedeh Maryam Hosseini, Saeed Shokuhi Rad, Mohammadreza Ansari Astaneh, Raheleh Mahboub Farimani

**Affiliations:** 1 Eye Research Center, Mashhad University of Medical Sciences, Mashhad, Iran; 2 Department of Medical Informatics, School of Medicine, Mashhad University of Medical Sciences, Mashhad, Iran; 3 Department of Medical Informatics, Kerman University of Medical Sciences, Kerman, Iran

**Keywords:** Artificial intelligence, Open globe injury, Visual acuity, Machine learning, Multi-class classification, Variables predictive of visual and surgical outcomes

## Abstract

**Introduction:**

Open globe injuries (OGI) represent a main preventable reason for blindness and visual impairment, particularly in developing countries. The goal of this study is evaluating key variables affecting the prognosis of open globe injuries and validating internally and comparing different machine learning models to estimate final visual acuity.

**Materials and methods:**

We reviewed three hundred patients with open globe injuries receiving treatment at Khatam-Al-Anbia Hospital in Iran from 2020 to 2022. Age, sex, type of trauma, initial VA grade, relative afferent pupillary defect (RAPD), zone of trauma, traumatic cataract, traumatic optic neuropathy (TON), intraocular foreign body (IOFB), retinal detachment (RD), endophthalmitis, and ocular trauma score (OTS) grade were the input features. We calculated univariate and multivariate regression models to assess the association of different features with visual acuity (VA) outcomes. We predicted visual acuity using ten supervised machine learning algorithms including multinomial logistic regression (MLR), support vector machines (SVM), K-nearest neighbors (KNN), naïve bayes (NB), decision tree (DT), random forest (RF), bagging (BG), adaptive boosting (ADA), artificial neural networks (ANN), and extreme gradient boosting (XGB). Accuracy, positive predictive value (PPV), recall, F-score, brier score (BS), Matthew correlation coefficient (MCC), receiver operating characteristic (AUC-ROC), and calibration plot were used to assess how well machine learning algorithms performed in predicting the final VA.

**Results:**

The artificial neural network (ANN) model had the best accuracy to predict the final VA. The sensitivity, F1 score, PPV, accuracy, and MCC of the ANN model were 0.81, 0.85, 0.89, 0.93, and 0.81, respectively. In addition, the estimated AUC-ROC and AUR-PRC of the ANN model for OGI patients were 0.96 and 0.91, respectively. The brier score and calibration log-loss for the ANN model was 0.201 and 0.232, respectively.

**Conclusion:**

As classic and ensemble ML models were compared, results shows that the ANN model was the best. As a result, the framework that has been presented may be regarded as a good substitute for predicting the final VA in OGI patients. Excellent predictive accuracy was shown by the open globe injury model developed in this study, which should be helpful to provide clinical advice to patients and making clinical decisions concerning the management of open globe injuries.

## Introduction

Open globe injury (OGI) is a potentially blinding ocular injury that is a full-thickness wound of the eye wall [1]. Globally, OGI exhibits an alarming annual incidence of nearly 203,000 cases [2], contributing substantially to permanent visual impairment and blindness [3]. In comparison to closed-globe injuries, OGI has a more visual damage, usually requires surgical repair, and increases the financial cost to society, the healthcare system, and patients. Numerous factors influence the final visual acuity (VA) in ocular trauma patients. Key determinants are age, trauma mechanism, whether relative afferent pupillary defects (RAPDs) are present or not, initial VA, hyphema, the wound’s size and location, intraocular foreign body (IOFB), vitreous hemorrhage, detachment of retinal, damage to the lens, and the Ocular Trauma Score (OTS) value, as highlighted by previous studies [4, 5].

From a clinical standpoint, accurately estimating a patient’s final visual outcome when an open globe injury occurs poses a significant challenge. To address this, establishing precise predictive models becomes imperative. The Ocular Trauma Score (OTS) is the most popular approach [6], which considers six factors, including the initial VA, endophthalmitis, retinal detachment, globe rupture, perforating injury, and relative afferent pupillary defect (RAPD) to provide prognostic assessments. However, OTS has been developed nearly 20 years ago. The improvements of surgical methods and equipment, especially the advancement of vitreoretinal surgeries during this period, probably has impacted the validity the OTS as a prognosis estimation system. Notably, OTS lacks consideration for certain complications of ocular trauma, such as traumatic cataract, which is a treatable condition [7]. In response to these limitations, alternative prognostic models have been proposed.

The classification and regression tree (CART), introduced in 2008, offers another approach for predicting visual outcomes in OGI patients [8]. Islam et al. and Toit et al. validated the OTS score to predict final VA and announced it as a valuable tool [9, 10]. By Compare both OTS and CART by Lee et al. and Man et al., Lee recommend that using both OTS and CART together in eye trauma assessments leads to more accurate predictions of vision outcomes while Man found OTS has higher accuracy [11, 12]. Choi et al. (2021) established a predictive tool for OGI utilizing machine learning algorithms on 171 patients, demonstrating the evolving landscape of prognostic methodologies. Boosted decision tree had the best result in predicting final VA [13–15]. Aoun et al. (2023) investigated the application of Support Vector Machines (SVM) and Neural Networks for predicting final visual acuity (VA) in 87 patients with ocular trauma. Their findings demonstrated superior accuracy with SVM compared to neural networks [16].

Algorithms leveraging machine learning exhibit robust capabilities in processing medical decision-making data, particularly in the realm of clinical predictions [17, 18]. developing a model able to forecast outcomes based on known outcomes and new input values, the supervised learning algorithm is utilized [19]. . This approach is particularly well-suited for handling extensive and intricate medical datasets [20].

In the context of ocular trauma, specifically Open Globe Injury (OGI), our study focused on harness the power of supervised machine learning approaches. Through the application of these methodologies, we endeavored forecasting the final Visual Acuity (VA) in patients affected by OGI.

## Materials and methods

### Study design and ethics

**Fig. 1:**
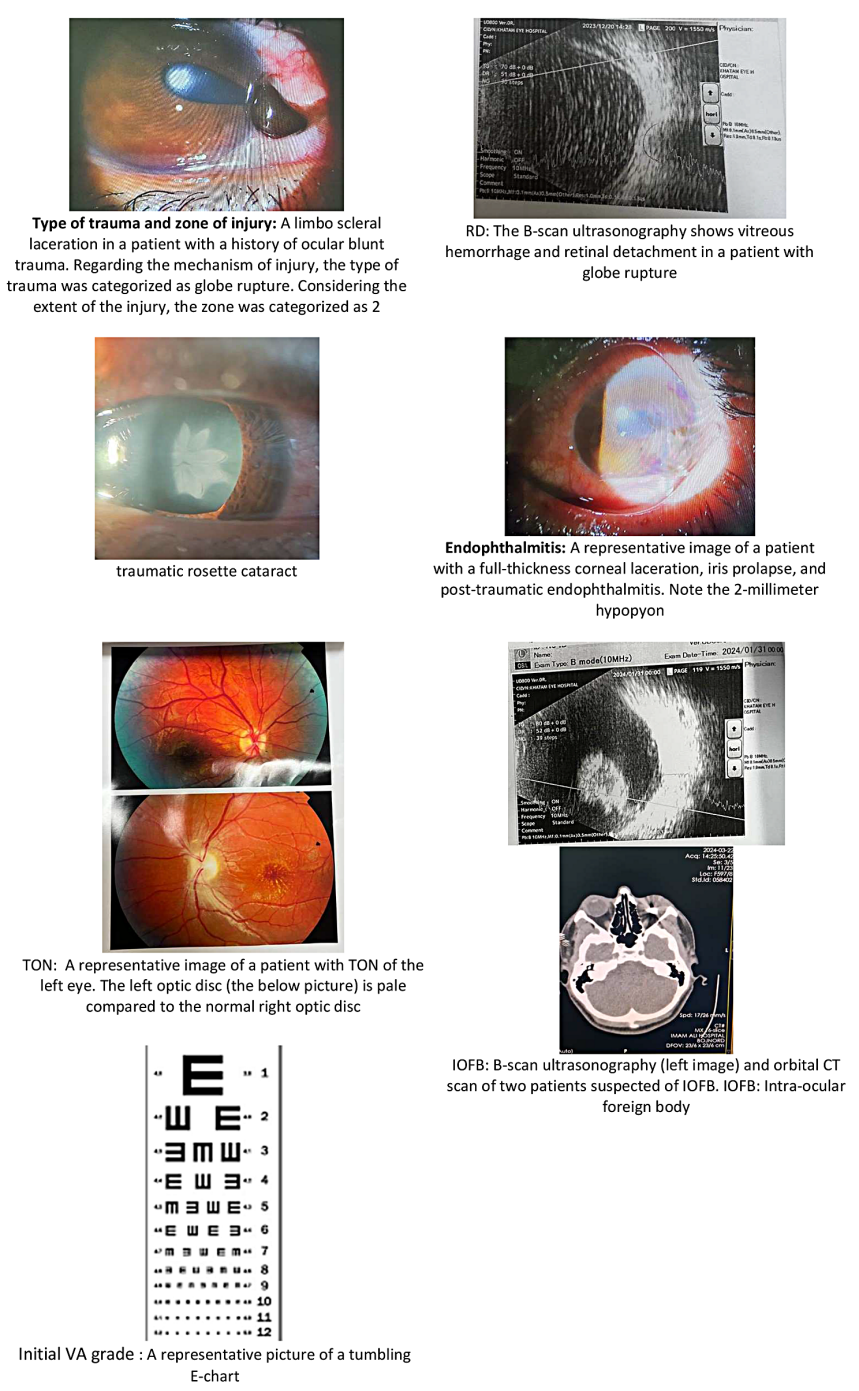
Images of dataset features

A comprehensive retrospective review was undertaken, involving 301 treated Open Globe Injuries (OGI) patients at Khatam-al-Anbia Eye Hospital, Mashhad, Iran during the period spanning 2020 to 2022. The Institutional Review Board of Mashhad University of Medical Sciences approved this study (IRB code: IR.MUMS.MEDICAL. REC.1399.060.). The study’s retrospective design ensured the meticulous examination of past cases while safeguarding the confidentiality and anonymity of the patients’ data. We declare that all techniques were developed in accordance with the regulations and rules.

i. Inclusion and Exclusion Criteria In this investigation, the study cohort comprised all admitted patients diagnosed with Open Globe Injuries (OGI), with no limitations based on age or gender. According to the implementation protocol in our center, OGI patients undergo primary repair surgery within 24 h of referral. Considering that the delay in restoration may affect the final vision prognosis if the surgery is delayed for more than 48 h, this case was not included in the study. Strict data cleaning techniques were used to guarantee the dataset’s dependability and integrity. A meticulous process was undertaken to eliminate noise, irrelevant information, and inconsistencies within the collected data. This involved the removal of null values, ensuring that missing or incomplete data did not compromise the quality of the analysis. Additionally, unrealistic values were identified and systematically excluded, further enhancing the accuracy and validity of the study findings.
ii. Input Data The data was collected from OGI dataset was included 301 patients with 12 features as prognostic factors including: Age, Sex, Type, Grade VA (Initial VA grade), Relative Afferent Pupillary Defect (RAPD), Zone, Traumatic Cataract, Traumatic optic Neuropathy (TON), Intraocular Foreign Body (IOFB), Retinal Detachment (RD), Endophthalmitis, and OTS grade. More descriptions and related images of dataset features are provided in Table 1; Fig. 1.

**Table 1:** A description of features selected for the models

Feature	Description
**Age**	The patients’ age was registered from their medical records.
**Initial VA grade**	We used a standard tumbling E-chart to measure the initial visual acuity. The reproducibility of VA assessment with standard charts was investigated previously [21].
**Type of trauma and zone of injury**	the mechanism of trauma determines the type of trauma that was assessed by history taking. In this study, the zone of injury was determined after careful evaluation of the sclera and cornea in the operating room and determining the extent and distance of the tear to the limbus in OGI patients.
**Relative Afferent Pupillary Defect (RAPD)**	To evaluate RAPD in OGI patients, this examination was performed with a standard method in a dark room with the help of a penlight with medium light. The result of Marcus Gunn’s pupil evaluation is recorded in the center’s emergency room after the approval of two ophthalmologists.
**Retinal Detachment (RD)**	The diagnosis of RD in this study was made with the help of clinical evaluation and ultrasound.
**Traumatic cataract**	Traumatic cataract is the crystallin lens opacification that occurs after a blunt or sharp ocular trauma.
**Endophthalmitis**	The diagnosis of post-traumatic endophthalmitis was made clinically in this study. The presence of an anterior chamber inflammation (hypopyon or fibrin reaction), vitritis, and retinitis were the main clinical indicators of post-traumatic infectious endophthalmitis.
**Traumatic optic Neuropathy (TON)**	Traumatic optic neuropathy occurs due to direct or indirect damage to the optic nerve caused by trauma to the eye or head. Clinical diagnosis is based on examination findings and history. Performing orbital CT, visual acuity measurement, visual field examination, and optic nerve head and peripapillary optical coherence tomography (OCT) imaging are methods that help to diagnose TON. In this center, the diagnosis of TON is made with the help of the mentioned methods and after confirmation by a neuro-ophthalmologist.
**Intraocular Foreign Body (IOFB)**	Clinical examination, ultrasonography, and computed tomography (CT-scan) were used to assess the patients suspected of having IOFB.

Within this dataset, a dual-feature categorization was implemented, classifying information into demographic and clinical parameters for each OGI patient. These categories were pivotal as input data for the comprehensive study.

Three different types of features for preprocessing and developing prognosis models were considered: (1) categorical features, (2) continuous features, and (3) binary variables (Table 2). Table 3 displays the features distribution.

**Table 2:** The classification and type of features selected for the models

Feature	Type	Classification
**Sex**	Binary	Male/Female
**Type of trauma**	Categorical	I: Rupture, II: Penetration, III: Perforation, IV: Other.
**Zone**	Categorical	I: Corneal injury, II: limboscleral laceration (5 mm from limbus), III: Scleral laceration (5 mm < from limbus)
**RAPD**	Binary	Yes/No
**Retinal Detachment (RD)**	Binary	Yes/No
**Traumatic Cataract**	Binary	Yes/No
**Endophthalmitis**	Binary	Yes/No
**Traumatic Optic Neuropathy (TON)**	Binary	Yes/No
**Intraocular Foreign Body (IOFB)**	Categorical	0: No IOFB, 1: one IOFB, 2: more than one IOFB, 3: IOFB resulting from an explosion
**Age**	Continuous	--
**Initial VA grade**	Categorical	I: VA ≥ 0.7, II: 0.7 > VA ≥ 0.1, III: 0.1 > VA ≥ 0.05, IV: VA: Hand motion detection, V: Light perception detection.
**OTS score Grade**	Categorical	OTS score: I, II, III, IV, V.

**Table 3:** The distribution of features for the models

Feature	Mean	Standard Deviation	Minimum	Maximum
**Sex**	1.8	0.4	1	2
**Type of trauma**	2.6	0.8	1	4
**Zone**	1.5	0.7	1	3
**RAPD**	0.3	0.5	0	1
**Retinal Detachment (RD)**	0.1	0.3	0	1
**Traumatic Cataract**	0.4	0.5	0	1
**Endophthalmitis**	0	0.1	0	1
**Traumatic Optic Neuropathy (TON)**	0	0.3	0	1
**Intraocular Foreign Body (IOFB)**	0.2	0.5	0	3
**Age**	25.1	17.0	1	74
**Initial VA grade**	3	1.3	0	10
**OTS score Grade**	3	1.2	1	5

iii. Outcome The final visual acuity (VA) is a continuous number between 0 and 1. The mean ± standard deviation of the duration of the follow-up was 364.5 ± 53.2 days with the range of 275–402 days. The final VA was defined as the patient’s best corrected distance VA at the last follow-up examination after performing rescue surgeries such as pars plana vitrectomy, cataract surgery etc. to manage early and late complications. In the pursuit of enhanced precision through machine learning methodologies, a pragmatic approach involves the categorization of this continuum into three distinct classes. Table 4 provides a visual representation of the stratification of final visual acuity within these classes. Obviously, binary outcome has more accuracy than multiclass outcome. This classification approach discerns patients into three categories based on their final visual acuity outcomes: those with poor VA (83 individuals, accounting for 27.5% of the cohort), moderate VA (84 individuals, constituting 27.9%), and good VA (134 individuals, representing 44.5% of the studied population).

**Table 4:** Categorized final visual acuity

Final Visual Acuity	Category	Number in the category
0–0.1	0 poor VA	83 (27%)
0.1–0.7	1 moderate VA	84 (28%)
0.7–1	2 good VA	134 (45%)

### Methodology

**Fig. 2:**
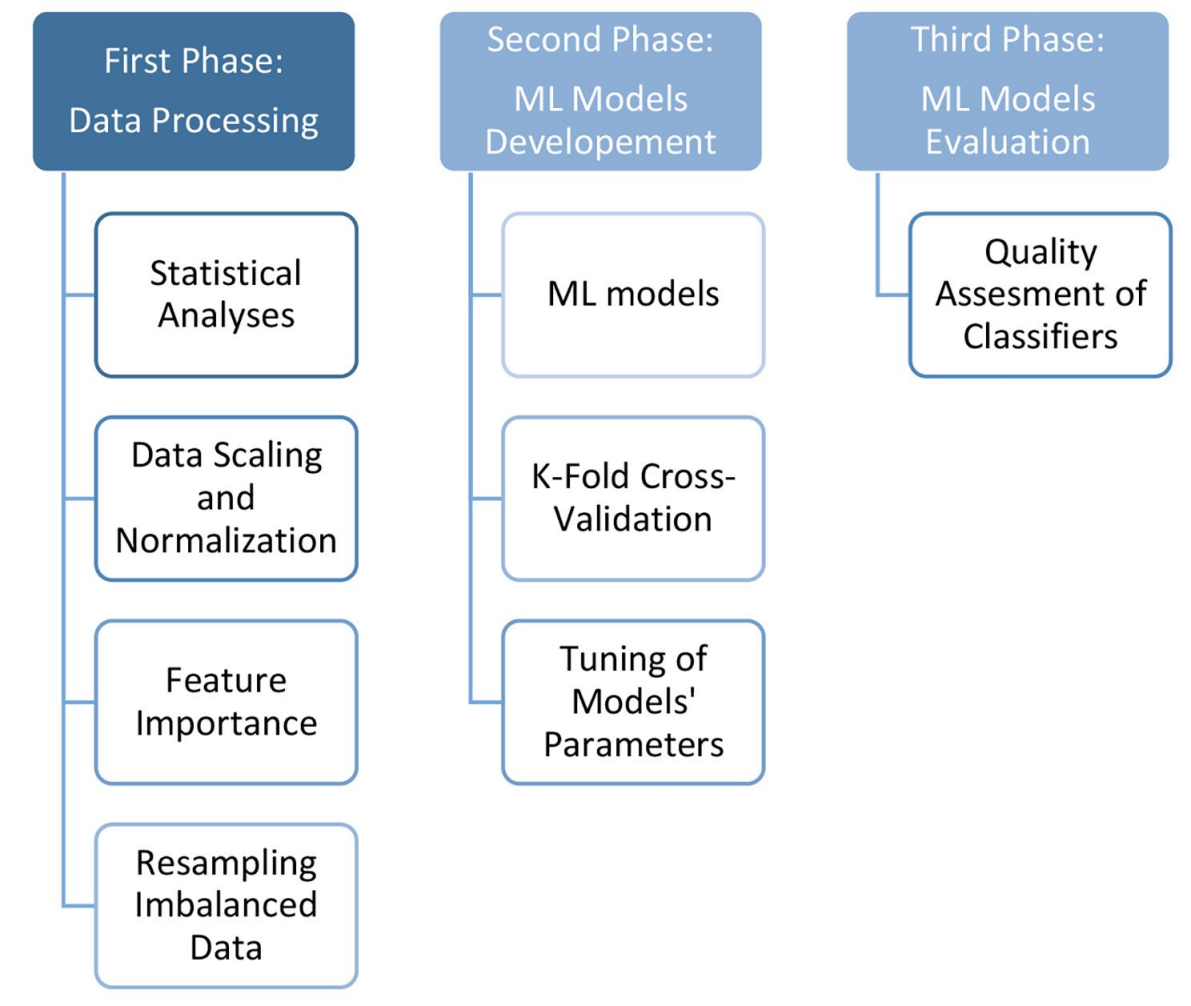
Three-Phases method for prediction final VA in OGI patients

This study presents a visual acuity prediction model. First of all, preprocessing and feature engineering on dataset’s features were done. After that, ten different ML models were developed. Finally, all developed models were assessed determining the optimal model based on outcome. Figure 2 illustrates the Three-Phases methods. The steps as follows.

#### Step one: data preprocessing and feature engineering

**Fig. 3:**
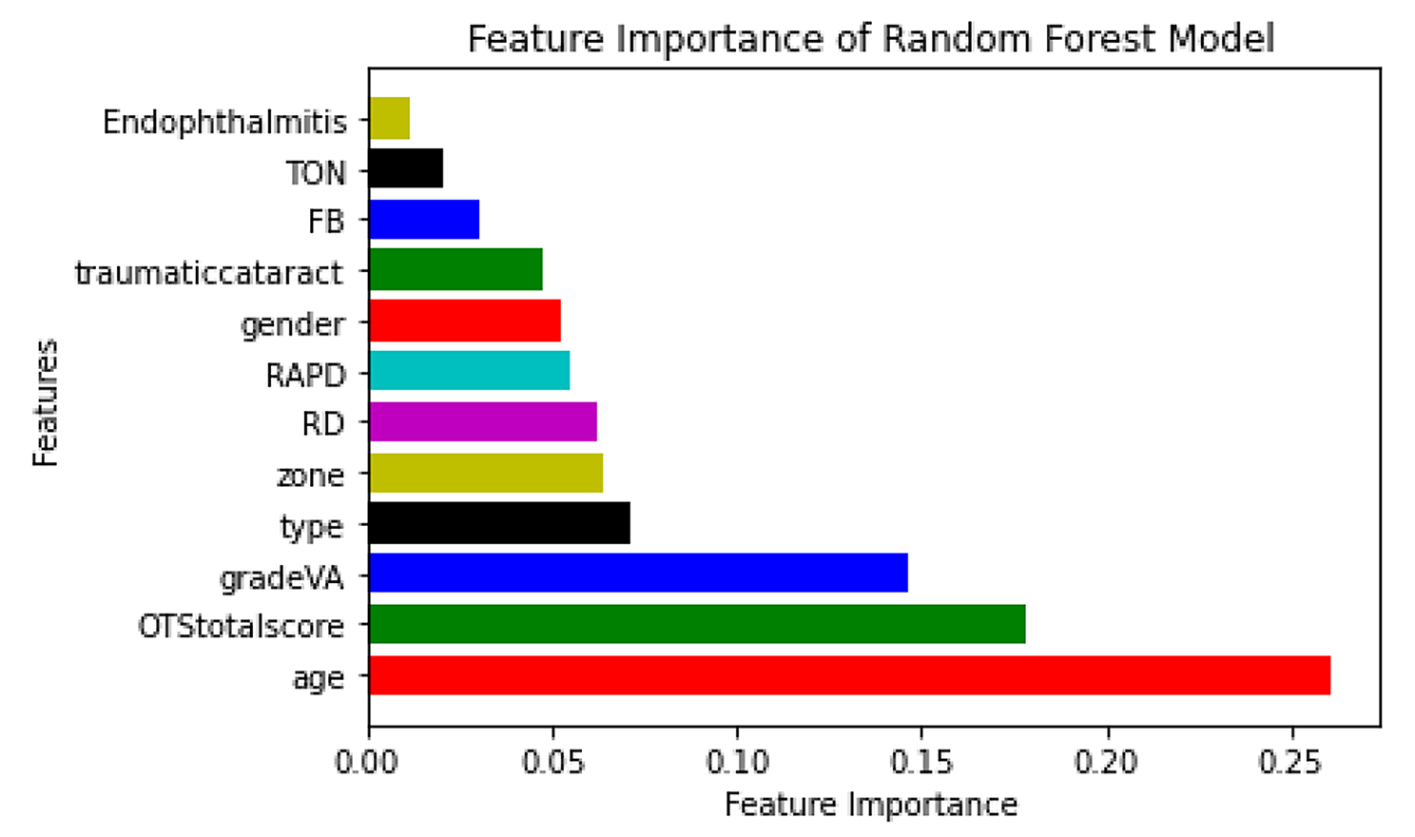
The importance of feature by Random Forest Model

There were four phases to preprocess data. After handling missing data and applying include/exclude criteria, these stages were followed: (1) Statistical analysis of input and output characteristics was done; (2) Data scaling, normalization techniques, and feature selection (the effect of features on predictions) were employed to handle different input data binary, categorical, and continuous); (3) To reduce the impact of unbalanced datasets, data sampling techniques were applied. Detailed sections as follows.

i. Statistical Analyses The features and outcomes were subjected to a descriptive analysis. The statistically significant differences between the values of categorical and continuous features in three classes (poor VA, moderate VA, and good VA) were compared using the Chi-square test and the t-test. Also, to remove any potential duplication, features and outcome correlations were calculated.
ii. Data Normalization and Scaling In order to reduce the effects of differing continuous and categorical feature value ranges on the ML models performance, data scaling methods were proposed. All categorical features including age and gender were normalized by one-hot encoding to zero and one. Also, numerical features were normalized by Z-score technique to bring them to a common scale.
iii. Feature Importance For feature selection, we employed mutual information and random forest techniques, which provide each feature a score, then arrange the features in order of that score. It is important to identify irrelevant attributes from our model. The most significant input variables for the final VA were chosen and imported to model. Results shows the accuracy with all features versus only important features has no significant difference, therefore, all features entered to model. Figure 3 shows feature importance.
iv. Resampling Imbalanced Data The imbalanced distribution of groups leads to overfit and poor performance of ML models [22]. This was one the most challenging problem in our dataset. Over-sampling and under-sampling sampling techniques were offered to deal with this problem [23]. For increasing sample counts in groups with low cases, Random Over-Sampling (ROS), Synthetic Minority Oversampling Technique (SMOTE), and borderline SMOTE are used, while for decreasing sample counts in groups with high cases, under-sampling algorithms, such as Random Under-Sampling (RUS) and Tomek links, were applied [24–27]. On the other hand, the SMOTE Tomek algorithm is a combination of oversampling and undersampling techniques [23]. . Moreover, the technique makes use of SMOTE for data enhancement on the minority class and Tomek Links to remove some samples from the majority class. This technique is better at improving the performance of machine learning models and eliminating noise or ambiguities in decision making. The current study assesses sampling strategies in the following categories: ROS, SMOTE, RUS, Tomek, and SMOTE Tomek using a basic LR model. In comparison with other methods, SMOTE Tomek has shown the best results.

#### Step two: development of ML models

We introduce our final strategy for OGI CDSS using cross-validation to determine the best model parameters and configurations in this section.

We were considered as the most indicative machine learning models with the best performance in prior studies as selected models to predict final VA in the OGI patients. K-fold cross-validation to train models and avoid the problem of overfitting was used in this study. We used cross-validation within the training set (80% of the data) to assess model performance and select optimal hyperparameters. By dividing the training data into folds, we could train models on one fold and evaluate them on the others, simulating unseen data [28]. . For ANN, the model employs a sequential architecture consisting of a single hidden layer with 16 neurons and ReLU activation, followed by an output layer with 3 neurons and softmax activation for multi-class classification. Hyperparameters were selected through a grid search methodology to optimize performance based on validation accuracy. The final choices include the RMSprop optimizer, a batch size of 10, and a maximum of 8,000,000 epochs. Early stopping was implemented to prevent overfitting, halting training if validation loss didn’t improve for 10 consecutive epochs. A K-Nearest Neighbors classifier with 5 neighbors was trained and evaluated. The number of neighbors acquired using K-folds to obtain a more robust estimate of optimal value. The Random Forest Classifier was tuned with grid search, exploring different numbers of trees (2-100), maximum tree depths [5–15], minimum samples for splitting (2-100), and minimum samples per leaf [1–5]. The Support Vector Machine used a linear kernel with a regularization parameter optimized through grid search. The Decision Tree Classifier had its maximum depth and minimum split/leaf samples similarly tuned. Random state of 42 used for reproducibility in ML models.

Performance metrics were used to assess K-fold cross-validation on the training dataset. The models with the greatest performance are the ultimate predictors.

#### Step three: evaluation of the models’ performance

The state-of-the-art references for multiclass classifiers and regressors were used as the basis for determining the evaluation metrics. The total number of patients that final VA was correctly anticipated is represented by a true positive (TP) and the total number of patients with wrong predicted final VA is indicated by a false negative (FN). Also, the total number of patients whose blindness was accurately predicted is shown by a true negative (TN) and the total number of patients misdiagnosed as having good VA is represented by a false positive (FP).

Recall, F1 score, Mathews Correlation Coefficient (MCC), Area Under Curve of Receiver Operator Characteristic (AUC-ROC), the Area Under the Curve of Precision-Recall (AUC-PRC), Accuracy, the Calibration Plot, and the Brier Score was computed [29–32].

## Results

In this study, the Python 3.8 (Anaconda/Jupyter) platform, along with the Pandas, Scikit-learn, and NumPy frameworks, were utilized in the development, evaluation, and visualization of all models. The machine, which was running Microsoft Windows 10 Enterprise, had a 2.5 GHz Intel Core i5 × 64 processor and 4 GB of RAM. The next subsection will provide the findings of the statistical examination of the input features and the significance of features in predictive models. Furthermore, the performance of models to predict final VA is evaluated.

**Table 5:** Baseline Clinical characteristics of OGI patients

Features		Poor VA 83 (27.6)	Moderate VA 84 (27.9)	Good VA 134 (44.5)	Total 301 (100)	*P*-Value
		N (%)	N (%)	N (%)	N (%)	
		Mean ± SD	Mean ± SD	Mean ± SD	Mean ± SD	
**Sex**	Male	64 (26.4)	80 (33.1)	98 (40.5)	242 (80.4)	< 0.001^*^
	Female	19 (32.2)	4 (6.8)	36 (61)	59 (19.6)	
**Type**	I	35 (83.3)	6 (14.3)	1 (2.4)	42 (14)	< 0.001^*^
	II	12 (26.1)	11 (23.9)	23 (50)	46 (15.3)	
	III	27 (13.5)	64 (32)	109 (54.4)	200 (66.4)	
	IV	9 (69.2)	3 (23.1)	1 (7.7)	13 (4.3)	
**Zone**	I	20 (11.3)	58 (32.8)	99 (55.9)	177 (58.8)	< 0.001^*^
	II	35 (41.2)	21 (24.7)	29 (34.1)	85 (28.2)	
	III	28 (71.8)	5 (12.8)	6 (15.4)	39 (13)	
**RAPD**	No	28 (14)	57 (28.5)	115 (57.5)	200 (66.4)	< 0.001^*^
	Yes	55 (54.4)	27 (26.7)	19 (18.8)	101 (33.6)	
**Retinal Detachment (RD)**	No	52 (19.5)	81(30.3)	134 (50.2)	267 (88.7)	< 0.001^*^
	Yes	31 (91.2)	3 (8.8)	0 (0)	34 (11.3)	
**Traumatic Cataract**	No	45 (26.3)	33 (19.3)	93 (54.4)	171 (56.8)	< 0.001^*^
	Yes	38 (29.2)	51 (39.2)	41 (31.5)	130 (43.2)	
**Endophthalmitis**	No	81 (27.7)	79 (27.1)	132 (45.2)	292 (97)	0.159^*^
	Yes	2 (22.2)	5 (55.6)	2 (22.2)	9 (3)	
**Traumatic Optic Neuropathy (TON)**	No	63 (22.8)	81 (29.3)	132 (47.8)	276 (91.7)	< 0.001^*^
	Yes	20 (80)	3 (12)	2 (8)	25 (8.3)	
**Foreign Body (FB)**	0	72 (27.9)	71 (27.5)	115 (44.6)	258 (85.7)	0.626^*^
	1	9 (23.1)	13 (33.3)	17 (43.6)	39 (13)	
	3	2 (50)	0 (0)	2 (50)	4 (1.3)	
**Age**		29 ± 17.1	26.6 ± 18.2	21.8 ± 15.6	25.1 ± 17	0.006^**^
**Grade VA**		3.8 ± 0.9	3.1 ± 0.8	2.2 ± 1.2	2.9 ± 1.2	< 0.001^**^
**OTS Grade**		1.7 ± 0.8	2.8 ± 0.7	3.8 ± 0.9	2.9 ± 1.2	< 0.001^**^

* Chi-Square, **Independent Samples Tests

### Results of the statistical analysis

The findings of a descriptive statistical analysis of continuous and categorical features are displayed in Table 5. Of 301 OGI patients were admitted in the Ophthalmology Department at the Khatam-al-Anbia hospital, 12 features were selected. For continuous features, we computed the mean and standard deviation (SD), while for categorical features as well as binary features, the number and percentage were calculated. Moreover, the independent samples t-test was used for continuous features and the Chi-square test was used for categorical features to determine whether there was a statistically significant difference between the three study classes (Poor VA, Moderate VA, and Good VA). As a result, between most features of the three classes there were statistically significant differences (95% confidence interval, P-value less than 0.05). Although, in the features including age, intraocular foreign body (IOFB), and endophthalmitis were not significantly different between the three classes.

**Fig. 4:**
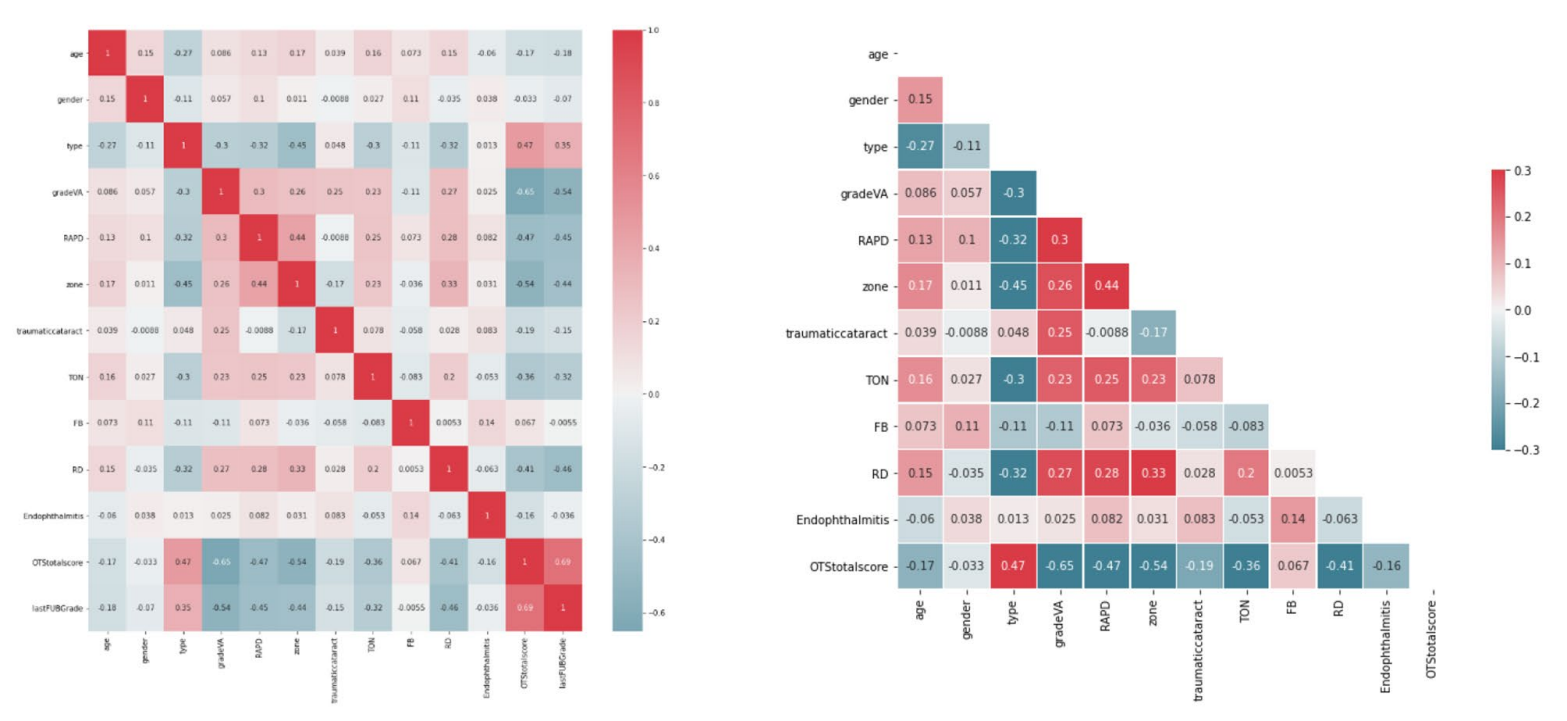
The pairwise correlation between features by Pearson test

Moreover, we calculated correlation between the factors. The relationship between these features is depicted in the correlation matrix. This correlation was better illustrated by the heat map. Evaluating the potential correlations between the features, the Pearson function with threshold 0.85 was used. The Pearson correlation coefficient can also be used to assess whether two variables have a significant relationship or not [33]. The corresponding cell is red if there is a strong correlation (greater than a threshold) between two features. The outcomes demonstrated that there is little correlation between the predictive features. The correlation of two features is indicated by the value inside each cell (refer to Fig. 4). According to the Fig. 4 characteristics such as age, OTS, Grade VA, and type are some of the essential features in OGI patients. To determine the clinical factors’ significance for the research, a random forest ranking is employed (Fig. 3). The most illuminating set of features can be chosen by plotting the random forest’s interpretation of feature importance. It selects many possible combinations of variables to find the best features [34]. Python 3.8 was used to draw all the figures.

### Evaluation of quality

Using a training dataset consisting of 80% of the primary records, we developed predictive ML models of OGI patients. Also, 80 and 20% of the dataset were randomly selected for training and testing. Moreover, the model parameters were tuned and determined using GridsearchCV, a technique for obtaining the best parameter values based on the grid’s specified set of parameters. The SMOTE Tomek method was employed to create a balanced dataset. Of the total, the group with good acuity made up 45% (*n* = 134), followed by the blind group (27%, *n* = 83) and the mild acuity group (28%, *n* = 84). The subsequent subsections assess the models’ predictive accuracy for the result.

#### Evaluation of classifiers quality to predict final VA for OGI patients

In this research the final VA in OGI patients predicted using ten models. A combination of classic and ensemble models, which consisted of Multinomial LR, KNN, SVM, ANN, Decision Tree, Random Forest, Naïve Bayas, XGB, Bagging, and ADA were used. The last four methods (Random Forest, XGB, Bagging, and ADA) are ensemble models. Then in the analysis phase, to compare the developed models three perspectives were applied: [1] predictive performance of models, [2] models’ ability assessment for discrimination employing the area under the curves (AUCs), and [3] a calibration plot to evaluate goodness-of-fit in models.

Predictive performance of models. Applying the measurements outlined in 2.3.3, the models’ performance was evaluated for predicting final VA of OGI patients. Table 6 displayed the suggested ML models’ predictive performance. Based on results, through all metrics, the ANN technique generated better results than any other model. It showed the highest values of AUC-ROC (0.96), AUC-PRC (0.91), precision (0.89), sensitivity (0.81), accuracy (0.93), F-measure (0.81), and MCC (0.75).

**Fig. 5:**
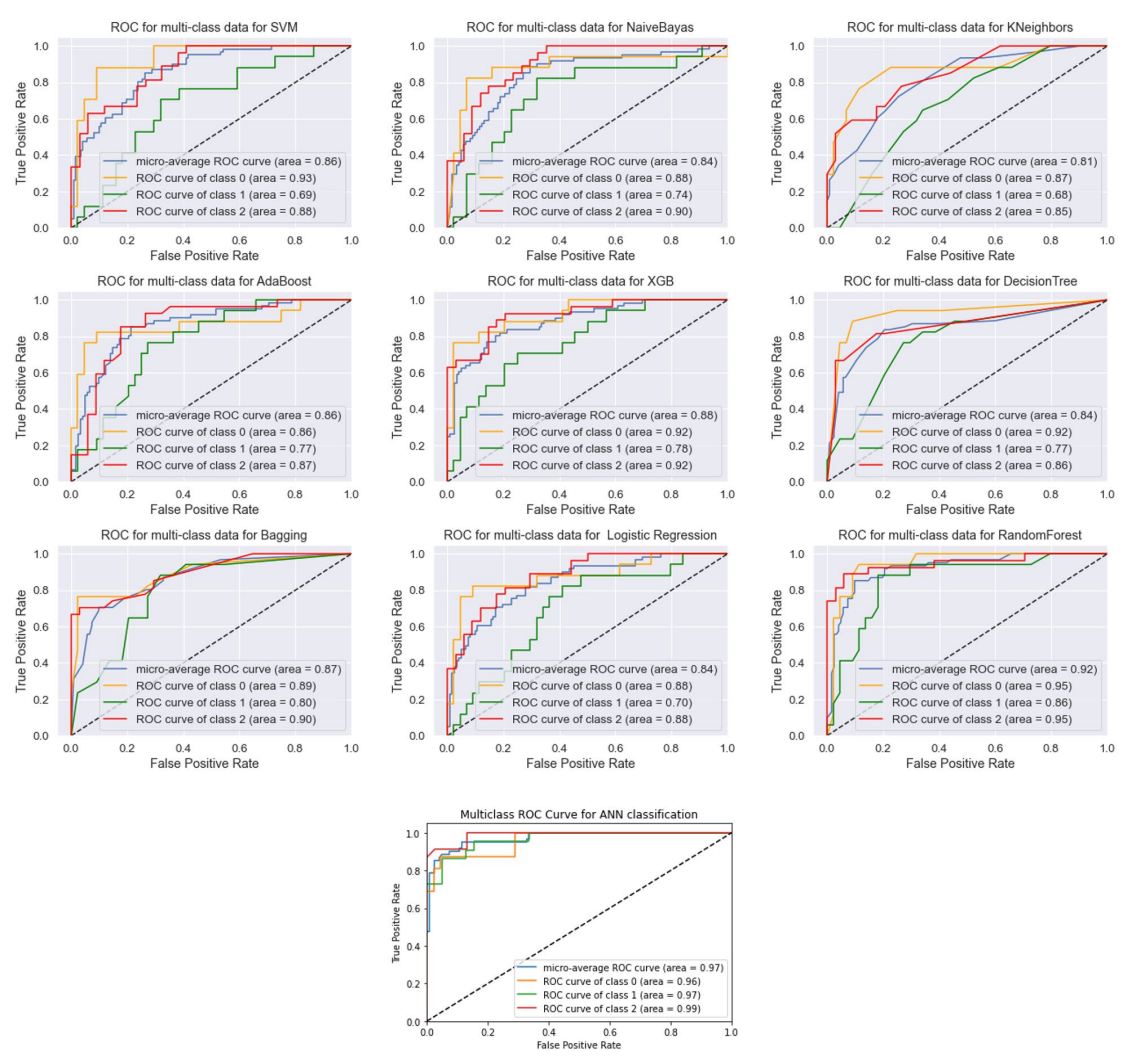
AUC-ROC for all models for each class

Assessment of models’ discriminating abilities. The receiver operating characteristic curves (AUCROC) and precision-recall curves (AUC-PRC) were applied evaluating the power of models to discriminate. While AUC-PRC displays precision values for recall (sensitivity) values, AUC-ROC illustrates the trade-off between specificity and sensitivity. Notably, for imbalanced datasets, metrics like AUC-PRC and AUC-ROC are usually considered to be the most informative [35]. Figure 5 illustrated AUC plots for ten models and for each class separately. AUC-ROC and AUC-PRC for all models in one shot illustrated in Fig. 6.1 and 6.2. AUC-ROC = 0.96 and AUC-PRC = 0.91, the highest overall value, were obtained by ANN, while other models showed respectable performance to forecast the final VA of OGI patients in both plots. Moreover, according to the accuracy of the methods, Repeated tests revealed that the models with the highest average accuracy were the ANN (Acc = 0.93) and the RF, LR, and XGB (Acc = 0.90).

Goodness-of-fit assessment in models. A visual tool for evaluating the degree of agreement between observations and predictions in the predicted values is the calibration plot. The Log loss, another metric for evaluating the quality of classification models, were calculated as calibrated log loss and uncalibrated log loss to compare, showed ANN (Calibrated-log-loss = 0.232) and RF (Calibrated-log-loss = 0.246) had smallest value in all models (Table 7). The calibration plots illustrated in Fig. 7. Also, the models with the best brier score loss values (BS, a metric composed of terms for refinement and calibration) among all of them were ANN (BS = 0.201) and RF (BS = 0.311) (Table 6).

**Table 6:** Predictive performance of classifiers for the final VA of OGI patients

Models Name	AUC-ROC	AUC-PRC	PPV	Sensitivity	Accuracy	F1	MCC	BS
SVM	0.86	0.75	0.812	0.764	0.885	0.787	0.709	0.433
Naïve Bayas	0.84	0.72	0.857	0.705	0.885	0.774	0.704	0.634
XGB	0.88	0.82	0.923	0.705	0.901	0.800	0.744	0.395
ADA	0.86	0.74	0.750	0.705	0.852	0.727	0.626	0.643
Bagging	0.89	0.79	0.764	0.764	0.868	0.764	0.673	0.412
Multinomial LR	0.84	0.75	0.866	0.764	0.901	0.812	0.748	0.439
KNN	0.81	0.69	0.750	0.705	0.852	0.727	0.626	0.476
Decision Tree	0.85	0.75	0.785	0.647	0.852	0.709	0.617	0.413
RF	0.92	0.86	0.866	0.764	0.901	0.812	0.748	0.311
ANN	**0.96**	**0.91**	**0.894**	**0.819**	**0.930**	**0.855**	**0.811**	**0.201**

* Area Under Curve of Receiver Operator Characteristic (AUC-ROC), Area Under Curve of Precision-Recall (AUC-PRC), Precision (PPV), Sensitivity (Recall), Accuracy (Acc), F-measure (F1), Matthew’s Correlation Coefficient (MCC), Brier Score (BS).

## Discussion

This study aimed to evaluate and compare the efficacy of different ML algorithms regarding the prediction of the final VA in patients with OGI. We used 12 features in 301 patients to train and evaluate ML models. Gender, type of trauma, zone of involvement, RAPD, presence of traumatic cataract, endophthalmitis, RD, traumatic optic neuropathy, IOFB, age, OTS score, and initial VA were the selected features for prognostication. To address the imbalanced distribution of classes (three classes here) which leads to overfitting and low performance of ML classes, SMOTE technique was used.

**Fig. 6:**
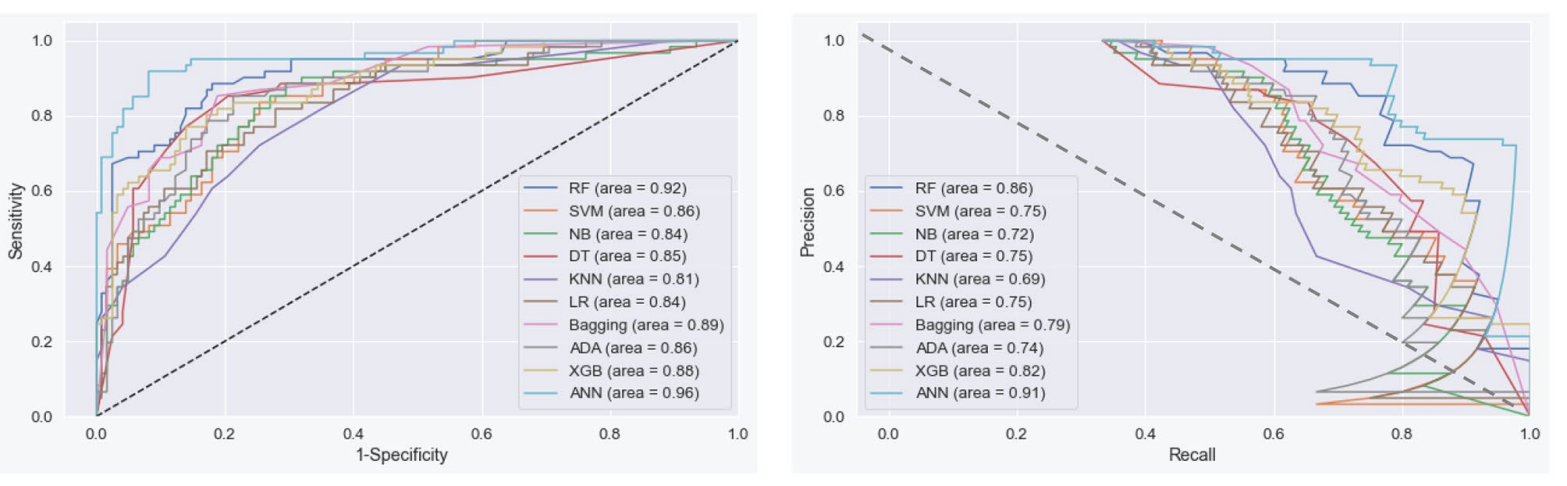
1) AUC-ROC 6.2) AUC-PRC

Since some variables were categorical and some were continuous, chi-square test and t-test were used to determine if there was a statistically significant difference between the three study groups (Poor VA, Moderate VA, and Good VA). In the features including age, intraocular foreign body (IOFB), and endophthalmitis were not significantly different between the three classes. The results of most of the previous studies indicate that the presence of IOFB or endophthalmitis is a significant factor in worsening the prognosis. Also, IOFB and endophthalmitis are indications for early vitrectomy (at the earliest possible time) in OGI patients [36]. This inconsistency could be due to early vitrectomy in these patients or the small sample size in each subgroup in this study. Moreover, we calculated correlation between the factors. The correlation matrix illustrates that the predictive features are not highly correlated. A random forest ranking is used to detect the importance of the clinical factors which showed that age, OTS, Grade VA, and type of trauma are some of the essential features in OGI patients. Choi et al. were applied some feature selection metrics [13] while others just used classical statistical methods. Factors associated with final VA which reported in previous studies includes age, initial VA, mechanism of injury, location and size of the wound, RAPDs, adnexal trauma, vitreous prolapse, and ocular tissue damage [6–8, 37–40]. Previous research on risk factors associated with prognosis in individuals with OGI has mostly used small sample sizes. Furthermore, the number of features was limited. Considering the complexities of treatment and the wide range of complications that may occur after OGI, the use of machine learning algorithms and various features can help in increasing the accuracy of predicting the vision prognosis of patients.

**Table 7:** Uncalibrated/ calibrated Log Loss of classifiers for the final VA of OGI patients

Models Name	Uncalibrated log loss	Calibrated log loss
**SVM**	0.716	0.586
**NB**	1.487	0.794
**DT**	3.802	0.584
**KNN**	0.800	0.734
**LR**	0.734	0.756
**Bagging**	3.289	0.262
**ADA**	0.999	0.733
**XGB**	0.733	0.328
**RF**	0.522	0.246
**ANN**	0.476	0.232

Employing a combination of classic and ensemble models, which consisted of Multinomial LR, KNN, SVM, ANN, Decision Tree, Random Forest, Naïve Bayas, XGB, Bagging, and ADA demonstrated ANN technique has superior results among all the models in all metrics. It shows the highest values of AUC-ROC (0.96), AUC-PRC (0.91), precision (0.89), sensitivity (0.81), accuracy (0.93), F-measure (0.81), and MCC (0.75). GridsearchCV as a technique for finding the optimal parameter values from a given set of parameters in a grid, was employed to tune and determine the model parameters during the 10-fold-cross-validation.

**Fig. 7:**
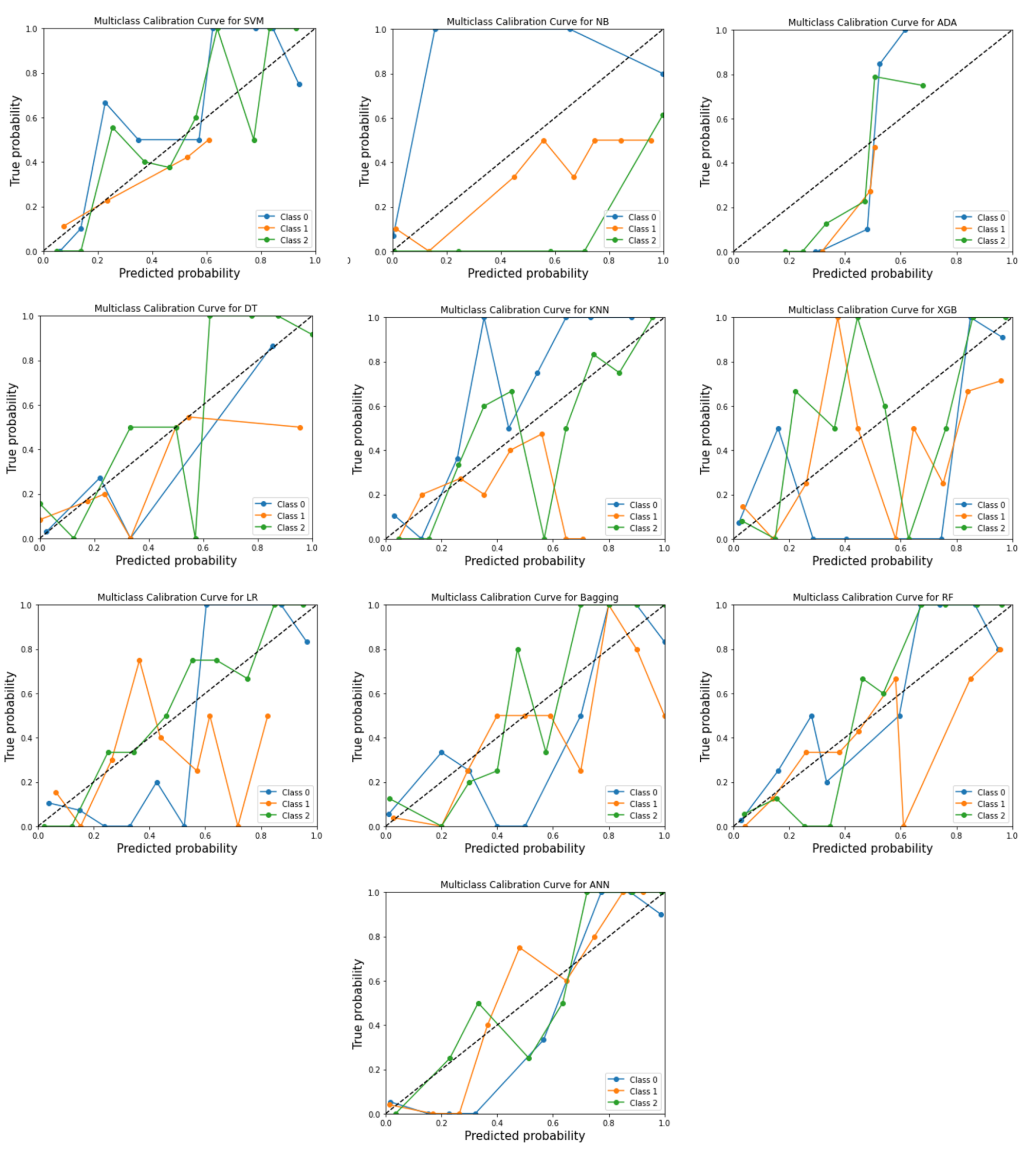
Models Calibration plots per each class to predict final VA in OGI patients

Successful initial repair and subsequent visual rehabilitation is a challenge for ophthalmic trauma surgeons. Besides, counseling of the trauma victim and his family is one of the crucial steps in the patient’s management. Despite the fact that the care of OGI has altered due to the development of new modalities and enhanced surgical techniques, we still need to counsel and prognosticate any patient with OGI before and after the primary repair surgery. Numerous studies have been evaluated the significant factors affecting final visual outcome in OGI previously [13, 41, 42]. In this study, a random forest ranking is used to detect the importance of the clinical factors. Age, OTS score, initial VA grade, and the type of trauma were the most important features. However, the reason for the less importance of features like IOFB and endophthalmitis can be the small number of patients with these conditions. Evaluation of the effect of age on the vision prognosis of OGI patients has had inconsistent results in previous studies [36, 43]. Inter-population variations in culture, lifestyle, mean lifespan, employment, and socioeconomic level might be the cause of this diversity. Regarding the type of injury, our results were compatible with those of previous studies. Globe rupture had the worst visual prognosis. Globe rupture is typically more closely linked to retinal detachment, optic nerve injury, and retinal damage. Furthermore, there are further challenges with primary repair surgery and rescue vitrectomy in this trauma mechanism. We showed that a poor initial visual acuity was an essential prognostic factor. According to this finding, lesser ocular tissue damage is reflected in a superior initial VA, which guarantees a better visual prognosis.

The study has some limitations and precautions. The data were collected from only one eye hospital from one city. Therefore, it is suggested that data be collected from several centers in different geographical locations, and external verification will have better performance and reliability. Moreover, the size of the dataset used was not significant, which is considered a precaution in this study. Besides, the occurrence of phthisis bulbi had not been investigated in this study. Future research topics could include evaluating the factors associated with the incidence of phthisis bulbi or sympathetic ophthalmia, selecting the weight corresponding to each feature and determining model parameters using meta-heuristic algorithms and fuzzy theory for ranking.

## Conclusion

As classic and ensemble ML models were compared, results shows that the ANN model was the best. As a result, the framework that has been presented may be regarded as a good substitute for predicting the final VA in OGI patients. Excellent predictive accuracy was shown by the open globe injury (OGI) predictive model developed in this research, which should be helpful to provide clinical advice to patients and to make clinical decisions concerning the management of open globe injuries.

## Data Availability

No datasets were generated or analysed during the current study.
